# The Effect of Orally Administered Probiotics on the Behavioral, Cellular, and Molecular Aspects of Adjuvant-Induced Arthritis

**DOI:** 10.32598/bcn.9.5.325

**Published:** 2018-09-01

**Authors:** Mahdi Shadnoush, Vida Nazemian, Homa Manaheji, Jalal Zaringhalam

**Affiliations:** 1. Department of Clinical Nutrition & Dietetics, School of Nutrition Sciences & Food Technology, Shahid Beheshti University of Medical Sciences, Tehran, Iran.; 2. Department of Clinical Nutrition, School of Nutrition and Food Sciences, Semnan University of Medical Sciences, Semnan, Iran.; 3. Neurophysiology Research Centre, Shahid Beheshti University of Medical Sciences, Tehran, Iran.; 4. Department of Physiology, School of Medicine, Shahid Beheshti University of Medical Sciences, Tehran, Iran.

**Keywords:** Probiotics, Hyperalgesia, Edema, Interleukin-1β (IL-1β), p38 Mitogen-Activated Protein Kinase (MAPK), μ-Opioid Receptor (MOR)

## Abstract

**Introduction:**

Rheumatoid Arthritis (RA) is a chronic autoimmune disease, which is accompanied with pain, hyperalgesia, and edema. Overproduction of pro-inflammatory cytokines and activation of intracellular signaling pathways sustain the RA symptoms considerably. There is a strong correlation between the expression of cytokines and opioid receptors in the arthritis process. Studies have shown that probiotics via different pathways such as reducing the levels of pro-inflammatory cytokines can alleviate inflammatory symptoms. Therefore, based on the crucial role of cellular and humoral immunity in induction of RA symptoms and potency of probiotics in modulation of immune responses, the purpose of this study was to investigate the effect of orally administered probiotics on the behavioral, cellular and molecular aspects of adjuvant-induced arthritis in male Wistar rats.

**Methods:**

Complete Freund’s Adjuvant (CFA)-induced arthritis was caused by single subcutaneous injection of CFA into the rat’s hind paw on day 0. Different doses of probiotics (1/250, 1/500 and 1/1000 [10^9^ CFU/g]) were administered daily (gavage) after CFA injection. Hyperalgesia, edema, serum IL-1β levels, μ-Opioid Receptor (MOR) expression, and p38MAPK (Mitogen-Activated Protein Kinase) activities were assessed on days 0, 7, 14 and 21 of the study.

**Results:**

The results of this study indicated the efficacy of probiotics in reducing hyperalgesia, edema, serum levels of Interleukin-1β, and p38MAPK pathway activity during different phases of arthritis as well as increasing the expression of MORs during chronic phase of CFA-induced arthritis.

**Conclusion:**

It seems that probiotics can effectively reduce inflammatory symptoms by inhibiting the intracellular signaling pathway and cytokine production.

## Highlights

- Effective dose of probiotics decreases inflammatory symptoms in Rheumatoid arthritis model.
- Effective dose of probiotics decreases interleukin-1β expression and p38MAPK activity in RA.
- Effective dose of probiotics increased Mu-opioid receptor expression in chronic phase of RA.

## Plain Language Summary

Pain is a highly unpleasant physical sensation caused by intense or damaging stimuli. The International Association for the Study of Pain (IASP) defines pain as “an unpleasant sensory and emotional experience associated with actual or potential tissue damage”. Rheumatoid arthritis is a chronic autoimmune disease which is characterized with edema, hyperalgesia, bone and cartilage destruction, and pain. Overproduction of proinflammatory cytokines and activation of intracellular signaling pathways have been strongly implicated in the generation of pathological pain states and induction of rheumatoid arthritis symptoms. It seems that modulation of central nervous system immunological responses, inhibition of proinflammatory pain, cytokines expression and intracellular signaling pathways activity could be a promising strategy for alleviating inflammatory pain symptoms. In this regard our results revealed that effective dose of probiotics could have anti-inflammatory effects and relieve inflammatory pain symptoms.

## 1. Introduction

Rheumatoid Arthritis (RA) is a systemic and chronic inflammatory autoimmune disease with unknown etiopathology which is characterized by pain, edema, hyperalgesia, and bone destruction (Nazemian, Nasseri, & Manaheji, & Zaringhalam 2016). CFA-induced arthritis is an inflammatory model widely used in physiopathologic and etiopathogenic drug studies because of its similarity to human RA. Intraplantar injection of CFA causes elevated firing of peripheral afferents in the spinal cord leading to hyperalgesia (Nasseri, Nazemian, Manaheji, & Zaringhalam, 2016).

Inflammation results in release of series of inflammatory mediators, and cytokines (Interleukin-1β [IL-1β]) and Tumor Necrosis Factor-α (TNFα) from damaged blood cells (Rodriguez-vita & Lawrence, 2010). Proinflammatory cytokines play an important role in pain modulation via interfering with nociceptive transduction, conduction and transmission (De Jongh et al., 2003). IL-1β has a key role in mediating auto-inflammatory diseases, joint destruction, and development of inflammatory symptoms, including hyperalgesia and edema (Dinarello, 2009). In addition, p38MAPKs (Mitogen-activated Protein Kinases) have a notable role in pro-inflammatory cytokine-induced signal transduction (De Jongh et al., 2003; Dinarello, 2009).

Activated p38MAPK (Phospho-p38MAPK) in the spinal cord is thought to play a key role in inflammation-induced spinal hyperalgesia, phosphorylation of transcription factors in the nucleus responsible for immediate-early genes regulation, provocation of other protein kinases, and mRNA stabilization (Zaringhalam, Akhtari, Eidi, Ruhani, & Tekieh, 2014). It seems that inhibition of proinflammatory cytokines like IL-1β and intracellular signaling pathways like p38MAPK could be a promising strategy to control inflammatory symptoms in RA (Korb et al., 2006; Moradi et al., 2014).

It is known that opioid receptors are involved in the pain modulatory system during inflammatory and hyperalgesic pain, and they could inactivate the neural pain fibers prototypically (Zaringhalam, Manaheji, Mghsoodi Farokhi, & Mirzaiee, 2008; Goldsmith, Uronis, & Jobin, 2011; Philippe et al., 2006). Zaringhalam et al. (2008) have shown that spinal μ-Opioid Receptors (MORs) mRNA expression increased significantly during 21 days of the study after CFA injection (Zaringhalam et al., 2008). On the other hand, TNF-α, IL-1β and Interferon gamma (IFN-γ) were able to induce MOR expression and subsequently, MOR agonists can reduce TNF-α and IL-1β production. Through regulation of cytokine production and modulation of T cell proliferation, MORs can exert its anti-inflammatory effects prototypically (Goldsmith et al., 2011; Philippe et al., 2006).

Probiotics are defined as selective nonpathogenic and antibiotic-resistant living microorganisms, including some commensal bacterial flora or yeasts belonging to the Lactobacillus, Bifidobacterium and Saccharomyces genera, which have diverse beneficial effects on host health and disease prevention and treatment. They have anti-mutagenic properties with few downsides (Shadnoush et al., 2013; Vanderpool, Yan, & Polk, 2008).

Probiotics can regulate immune responses by elevating the anti-inflammatory cytokines, for example, IL-10 which suppresses the Th1 responses, blocks the proinflammatory cytokines production, and modulates the intracellular signaling pathways including AKT, MAPK, and NF-κB. However, little is known about the effects of probiotics on regulation of MORs and their mechanisms of action (Vanderpool et al., 2008; Vaghef-Mehrabany et al., 2014; Samuel et al., 2008). Probiotics due to their immuno-modulatory and anti-inflammatory properties are able to reduce the symptoms of RA (Vaghef-Mehrabany et al., 2014).

Given the importance of effective treatments with fewer side effects using the anti-inflammatory and immunomodulatory properties of probiotics, this study aimed to examine the role of oral administration of probiotic on hyperalgesia, edema, IL-1β serum level, spinal p38MAPK activity, and MOR expression during different stages of adjuvant-induced arthritis in male Wistar rats.

## 2. Methods

### 2.1. Laboratory animals

In this research, adult male Wistar rats (n=168) weighing 200–220 g were selected for the study. These rats were housed in polypropylene cages under standard hygienic environmental conditions (22±2°C, humidity 60%–70%, and 12:12 h light:dark cycle). All animals, except the time during experiments, had access to standard food and water. (Zimmermann, 1983). For determining the effect of different dosage of probiotics on inflammatory pain model and the effectiveness of this treatment, a series of experiments were executed. Rats were randomly divided into seven experimental groups: 1. CFA group; 2. CFA control group; 3. CFA+vehicle group (CFA+Vehi); 4. CFA+1/250 probiotic group (CFA+Pb1); 5. CFA+1/500 probiotic group (CFA+Pb2); 6. CFA+1/1000 probiotic group (CFA+Pb3), and 7. Positive control group (CFA+ Indomethacin [Indo]) (Zaringhalam et al., 2016). Each group was divided into four subgroups based on different time points (days 0, 7, 14, and 21), each containing 6 rats.

### 2.2. Experimental procedure

In CFA group, arthritis was caused by single subcutaneous injection of (100μL) CFA containing heat-killed Mycobacterium tuberculosis suspended in sterile mineral oil (10 mg/mL; CFA; Sigma, St Louis, MO, USA) into the rat’s right hind paw on day zero (under light anesthesia with methoxy isoflurane). The CFA control group received sterile mineral oil only (100 μL), and the CFA+Vehi group received CFA+distilled water. Distilled water was administered after CFA injection by gavage once a day. The positive control group received indomethacin on a daily basis by gavage (5 mg/kg).

The treatment was a combination of probiotic bacteria strains such as Bifidobacterium breve, Lactobacillus casei, Lactobacillus bulgaricus, Lactobacillus rhamnosus and Lactobacillus acidophilus with 109 CFU/g (colony-forming units per gram) prepared at laboratory scale at the Physiology Department of Shahid Beheshti University of Medical Sciences in Tehran, Iran. Different dosages (4×10^6^ CFU/g, 2×10^6^ CFU/g and 10^6^ CFU/g for 1/250, 1/500 and 1/1000 dosages, respectively) were prepared by dissolving probiotics in distilled water, and from these prepared solutions, 1 mL was administered via gastric gavage on a daily basis from the first day after CFA injection up to day 21 (Shadnoush et al., 2013).

In this study, considering our previous studies, the molecular parameters (spinal p38MAPK, Pp38MAPK, MOR and β-actin), behavioral test (hyperalgesia), and paw edema and serum IL-1β levels were assessed on days 0 (before CFA injection), 7 (inflammatory phase), 14, and 21 (arthritic phase) (Zaringhalam et al., 2014).

### 2.3. Assessment of complete Freund’s adjuvant-induced arthritis and paw edema

To confirm the correct measurement of CFA injection, the volume of both paws before and after injection was tested at different time points. In brief, the rat’s hind paw was submerged to the tibiotarsal joint, into a transparent chamber of plethysmometer containing an electrolyte solution and paw edema volume was measured by displacement of an electrolyte solution in a plethysmometer chamber (Model 7141; Ugo Basile, Comerio-Varese, Italy). The volume of displacement was equal to the paw volume and measurement was conducted twice for each paw and then, the average was calculated (Zaringhalam et al., 2008).

### 2.4. Behavioral test (thermal hyperalgesia assessment)

Paw Withdrawal Latency (PWL) in response to radiant heat by plantar test was performed in all groups (Ugo Basilar, Verse, Italy). Rats were placed in Plexiglas chambers 30 minutes before the test in order to habituate to the test environment. Infrared light was projected to the hind paw and PWL was recorded. A cut-off time of 20 seconds was set to avoid paw injury. Hyperalgesia was performed three times for each paw at an interval of 5–10 minutes and then, the mean latency of the withdrawal responses for each paw was calculated. The mean of injected paw was subtracted from the other paw, and the obtained value represented the hyperalgesia in the injured paw (Nazemian et al., 2016).

### 2.5. Blood sampling and serum interleukin-1β measurements

Rats from all experimental groups were moderately anesthetized with methoxy isoflurane, and retro-orbital blood samples were collected in tubes. First and foremost, blood samples were centrifuged and then, serum levels of IL-1β were measured by using an enzymelinked immunosorbent assay kit (Bender MED System, UK) according to the manufacturer’s instructions. Blood serum was added to 96-well microplates which were coated with rat IL-1β-specific polyclonal antibody.

After incubation at room temperature (for 120 min) and two times washing, enzyme-linked polyclonal antibody specific for IL-1β was added to each well. Afterwards, color reagents were added after 120 min incubation at room temperature and re-washing. The intensity of color was measured by microplate reader (450 nm). A standard curve was made and the serum levels of IL-1β (pg/mL) in all samples were calculated (Bressan, Cunha, & Tonussi, 2006; Zaringhalam et al., 2016).

### 2.6. Spinal cord tissue extraction

In order to identify the expression of spinal p38MAPK, Pp38MAPK and MOR in each group, the lumbar segments of the rats’ spinal cord (L1–L5) were removed. For this purpose, rats were anesthetized with methoxy isoflurane, their heads removed, and the lumbar segment of spinal cord immediately dry-ice frozen and kept in liquid nitrogen and then, maintained for western blotting at −80°C (Zaringhalam, Tekieh, Manaheji, & Akhtari, 2013).

### 2.7. Evaluation of p38 mitogen-activated protein kinase activity and protein expression of μ-opioid receptor in western blot

Samples in lysis buffer containing Tris-HCl (pH=7.4), EDTA, NP-40 1%, NaCl, aprotinin, leupeptin, and PMSF were homogenized and then centrifuged. Protein extracts or supernatants were harvested for analysis of protein concentration by Bradford method. After diluting proteins with sample buffer, each cocktail was loaded in a lane and run on 12.5% Sodium Dodecyl Sulfate-Polyacrylamide Gel Electrophoresis (SDS-PAGE). Afterwards, separated proteins were transferred onto Immobilon PVDF membranes (Millipore, Bedford, MA, USA) using the Mini-Protean II (Bio-Rad).

Incubation with blocking buffer helped block nonspecific binding sites on the PVDF membrane. Next, the membrane was incubated with primary antibodies (Anti-Pp38 antibody, ab31828; Anti-P38 antibody, ab32557; Anti-MOR, ab10275 and β-actin, ab8227) and secondary antibodies (Anti-mouse antibody for Pp38 and antirabbit antibody for others) which were diluted in blocking buffer (Abcam, CA).

Immunoreactivity of proteins on the PVDF membrane was detected by chemiluminescence detection system (ECL, Amersham). Band intensities were assessed by densitometric analysis using Image-J software. For evaluating the spinal p38MAPK activation variations and spinal MOR expression, the ratio of Pp38/p38MAPK and MOR/β-actin were calculated (Zaringhalam et al., 2013).

### 2.8. Statistical analysis

The obtained data were expressed as the mean±SEM. For comparison of variants within groups, repeated measures ANOVA (1-way ANOVA) and post-hoc Tukey test were used. Also, for more accurate comparison of variants between groups on the same days, unpaired Student’s t test was used.

## 3. Results

### 3.1. Paw hyperalgesia variations during different phases of arthritis

**Figure 1.**
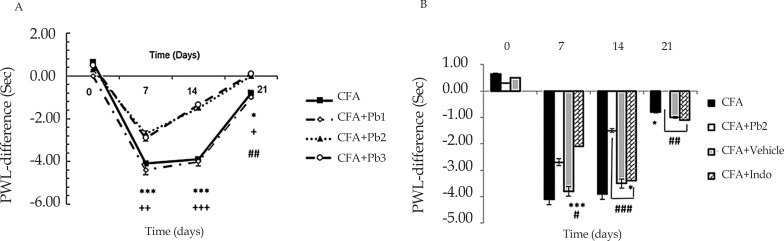
Hyperalgesia assessment A: Administration of different doses of probiotics could affect hyperalgesia. Results were expressed as Mean±SEM and n=6/group. ^*^ P≤0.05; ^**^ P≤0.01; and ^***^ P≤0.001: Comparison of hyperalgesia on days 7, 14 and 21 in the CFA group with baseline. + P≤0.05, ++ P≤0.01, and +++ P≤0.001: The difference in hyperalgesia on days 7, 14 and 21 compared with the same days in CFA and CFA+Pb2 (1/500) groups. ## P≤0.01: Comparison of hyperalgesia between days 14 and 21 in CFA group. B: Administration of effective dose of probiotics could reduce hyperalgesia. Results were expressed as Mean±SEM and n=6/group. * P≤0.05 and *** P≤0.001: Comparing the changes of hyperalgesia between CFA and CFA+Indo groups during different days of the study. # P≤0.05; ## P≤0.01; and ### P≤0.001: Comparing the changes of hyperalgesia between CFA+Indo and CFA+Pb2 (1/500) groups during different days of the study.

The results showed that hyperalgesia significantly increased on day 7 after CFA injection compared to the baseline (P≤0.01). But, hyperalgesia significantly decreased on days 14 and 21 compared to that on day 7 in the CFA group (P≤0.01). No significant differences were observed in the CFA control group during different days of study, compared to the baseline (results not shown). Oral administration of probiotics with 1/250 dose in the CFA+Pb1 group had no significant effect on hyperalgesia during different days of study, compared to the CFA group. Administration of probiotic with 1/500 and 1/1000 dose in the CFA+Pb2 and CFA+Pb3 groups significantly reduced hyperalgesia in different days of study compared to the same days in the CFA group (P≤0.05 for day 21, P≤0.001 for day 14 and P≤0.01 for day 7) (Figure 1A).

Administration of indomethacin significantly reduced hyperalgesia in the CFA+Indo group compared to the CFA group (P≤0.001 for day 7, and P≤0.05 for day 14). Hyperalgesia significantly increased in the CFA+Indo group on day 21 compared to the CFA group (P≤0.05). Moreover, the effective dose of probiotics (1/500) in the CFA+Pb2 group significantly reduced hyperalgesia on days 14 and 21 compared to the CFA+Indo group (P≤0.01 for day 21, and P≤0.001 for day 14). Furthermore, hyperalgesia on day 7 in the CFA+Indo group significantly decreased compared to the CFA+Pb2 group (P≤0.05). The changes in hyperalgesia in the CFA+Vehi group showed no significant differences with those in the CFA group (Figure 1B).

### 3.2. Paw edema variations during different phases of arthritis

**Figure 2.**
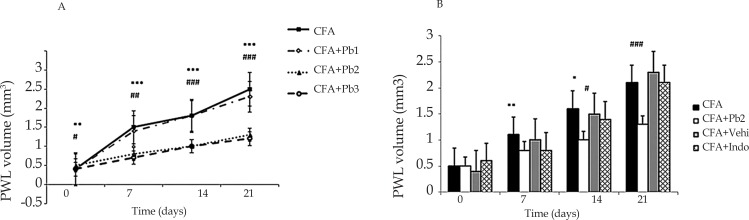
Paw volume variations A: Administration of different doses of probiotics could affect paw edema. Results were expressed as Mean±SEM and n=6/group. ^***^ P≤0.001: Comparison of edema in different days in the CFA group with baseline. ## P≤0.01 and ### P≤0.001: The difference in paw edema in different days of the study compared with the same days in CFA and CFA+Pb2 (1/500) groups. B: Administration of effective dose of probiotics could reduce paw edema. Results were expressed as Mean±SEM and n=6/group. ^*^ P≤0.05 and ^**^ P≤0.01: Comparing the changes of paw edema between CFA and CFA+Indo groups during different days of the study. # P≤0.05 and ### P≤0.001: Comparing the changes of paw edema between CFA+Indo and CFA+Pb2 (1/500) groups during different days of the study.

CFA injection into the rat’s hind paw induced edema which continued up to day 21 (P≤0.001). No significant differences were observed in the paw edema in different days of study compared to the baseline in the CFA control group (results not shown). The results showed that oral administration of probiotics with a dosage of 1/250 had no significant effect on edema in the CFA+Pb1 group compared to the CFA group during different days of study. Oral administration of probiotics with a dosage of 1/500 and 1/1000 in the CFA+Pb2 and CFA+Pb3 groups significantly reduced paw edema in different days of study compared to the same days in the CFA group (P≤0.01 for day 7, and P≤0.001 for days 14 and 21) (Figure 2A).

Administration of indomethacin significantly reduced paw edema in the CFA+Indo group on days 7 and 14 in comparison with the CFA group (P≤0.05 for day 14, and P≤0.01 for day 7). Comparison of the two groups of CFA+Pb2 and CFA+Indo showed that the effective dose of probiotics (1/500) significantly reduced paw edema on days 14 and 21 compared to the same days in the CFA+Indo group (P≤0.001 for day 21 and P≤0.05 for day 14). Paw edema in the CFA+Vehi group showed no significant difference compared to the CFA group (Figure 2B).

### 3.3. Serum interleukin-1β levels during different phases of arthritis

Serum levels of IL-1β increased in the CFA group in different days of study compared to that on day 0 (P≤0.001). Administration of mineral oil as a CFA solvent in the CFA control group had no significant effect on the serum levels of IL-1β (results not shown). Probiotics changed the serum levels of IL-1β in the CFA+Pb groups in a dose-dependent manner. Oral administration of probiotics with 1/250 dose had no significant effect on the serum IL-1β levels in the CFA+Pb1 group compared to the CFA group.

**Figure 3.**
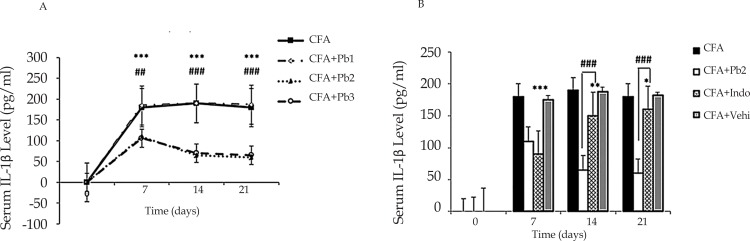
IL-1ß expression A: Administration of different doses of probiotics could affect serum levels of interleukin-1β. Results were expressed as Mean±SEM and n=6/group. ^***^ P≤0.001: Comparison of serum levels of interleukin-1β in CFA group compared with baseline in different days of the study. ## P≤0.01; and ### P≤0.001: Comparison of serum levels of interleukin-1β on days 7, 14 and 21 in CFA+Pb2 (1/500) group with CFA group. B. Administration of effective dose of probiotics could reduce serum levels of interleukin-1β. Results were expressed as Mean±SEM and n=6/group. ^*^ P≤0.05; ^**^ P≤0.01; and ^***^ P≤0.001: Comparison of serum levels of interleukin-1β between different days of the study in CFA+Indo group with CFA group. ### P≤0.001: Comparison of serum levels of interleukin-1β between different days of the study in CFA+ Indo group with CFA+Pb2 (1/500) group.

Oral administration of probiotics with 1/500 and 1/1000 doses significantly reduced IL-1β levels in the CFA+Pb2, and CFA+Pb3 groups compared to the CFA group (P≤0.01 for day 7 and P≤0.001 for days 14 and 21). There was no significant difference in serum levels of IL-1β between the two groups receiving probiotics with doses of 1/500 and 1/1000. Therefore, the probiotic dose of 1/500 was selected as the effective dose in this phase (Figure 3A).

Administration of indomethacin significantly reduced serum levels of interleukin-1β in the CFA+Indo group (P≤0.05 for day 21, P≤0.01 for day 14, and P≤0.001 for day 7). Comparison of the two groups, CFA+Pb2 and CFA+Indo showed that the effective probiotic dose (1/500) could significantly reduce the serum levels of interleukin-1β on days 14 and 21 compared to the same days in the CFA+Indo group (P≤0.001). The changes in the IL-1β levels in the CFA+Vehi group showed no significant difference with those in the CFA group (Figure 3B).

### 3.4. Variations in spinal p38 mitogen-activated protein kinase enzyme activity during different phases of arthritis

In order to detect the activity of the spinal p38MAPK enzyme, Pp38MAPK monoclonal antibody was used. Protein expression of p38MAPK indicated similar bands at the molecular masses of about 42 kDa in the spinal cord tissues of all experimental groups. The p38MAPK was considered as a loading control protein. Hence, for normalizing the differences in proteins loading, all data were expressed as the Pp38/p38MAPK ratios. Densitometric data from western blots demonstrated that phosphorylation of p38MAPK enzyme in the spinal cord of rats in CFA group significantly increased in different days of study compared to that on day 0 (P≤0.001).

**Figure 4.**
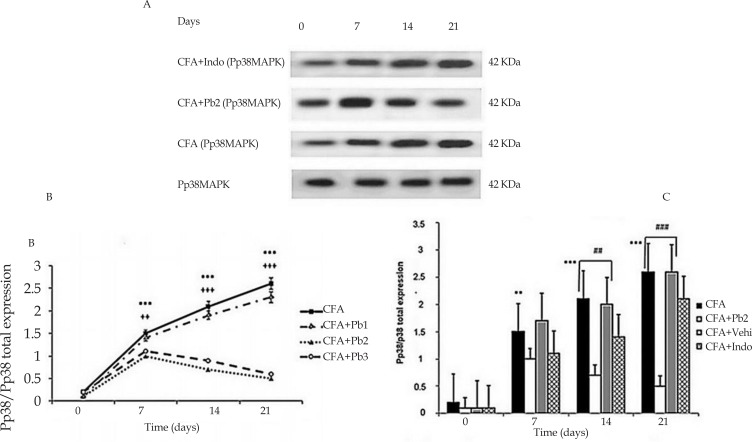
P38MAPK expression A. Immunoblots of spinal p38MAPK enzyme expression throughout different stages of arthritis. All data are represented as Mean±SEM (n=6/group). B. Administration of different doses of probiotics could affect Pp38/p38MAPK ratio. Results were expressed as Mean±SEM and n=6/group. ^***^ P≤0.001: Comparison of spinal p38MAPK protein band intensity in different days of study compared with baseline in CFA group. ++ P≤0.01; and +++ P≤0.001: The differences in the p38MAPK activity in the CFA+Pb2 (1/500) group on days 7, 14 and 21 compared with the same days in the CFA group. C. Administration of effective dose of probiotics could reduce Pp38/p38MAPK ratio. Results were expressed as Mean±SEM and n=6/group. ^**^ P≤0.01 and ^***^ P≤0.001: Comparison of Pp38/P38 MAPK ratios between different days of the study in CFA+Indo group with CFA group. ## P≤0.01 and ### P≤0.001: Comparison of Pp38/P38 MAPK ratios between different days of the study in CFA+Indo group with CFA+Pb2 (1/500) group.

Sterile mineral oil injection to the rat’s hind paw in the CFA control group did not cause considerable changes in the spinal Pp38/p38MAPK activity compared to the baseline in different days of study (graphing results not presented) (Figure 4A).

The results of this study illustrated that oral administration of probiotics with a dose of 1/250, had no significant effect on the activity of spinal Pp38/p38MAPK in the CFA+Pb1 group compared to the CFA group in different days of study. Moreover, oral administration of probiotics (1/500 and 1/1000) significantly diminished the activity of Pp38/p38MAPK in the CFA+Pb2, and CFA+Pb3 groups compared to the CFA group (P≤0.01 for day 7 and P≤0.001 for days 14 and 21). There was no significant difference in Pp38/p38MAPK activity between the two groups receiving probiotics with 1/500 and 1/1000 doses.

In this regard, the probiotic dose of 1/500 was selected as the effective dose in this phase (Figure 4B). Furthermore, administration of indomethacin significantly reduced p38MAPK activity in the CFA+Indo group (P≤0.01 for day 7 and 21, P≤0.001 for day 14). Comparison of the two groups, CFA+Pb2 and CFA+Indo showed that the effective dose of probiotics (1/500) could significantly reduce the activity of Pp38/p38MAPK on days 14 and 21 compared to the same days in the CFA+Indo group (P≤0.01 for day 14, and P≤0.001 for day 21). The changes in the p38MAPK activity in CFA+Vehi group had no significant difference with those in the CFA group (Figure 4C).

### 3.5. Variations in spinal μ-opioid receptor expression during different phases of arthritis

Spinal MOR expression illustrated similar band intensities at the molecular masses of about 75 kDa in the spinal cords of rats in all experimental groups. The β-actin was considered as a loading control protein for MOR. It is worthy of note that for normalizing the differences in loading of proteins, all data were stated as the MOR/β-actin ratios. Densitometric data from western blots revealed that spinal cord expression of MOR in CFA group increased considerably on days 14 and 21 compared to that value on day 0 (P≤0.01 for day 14, and P≤0.001 for day 21).

**Figure 5.**
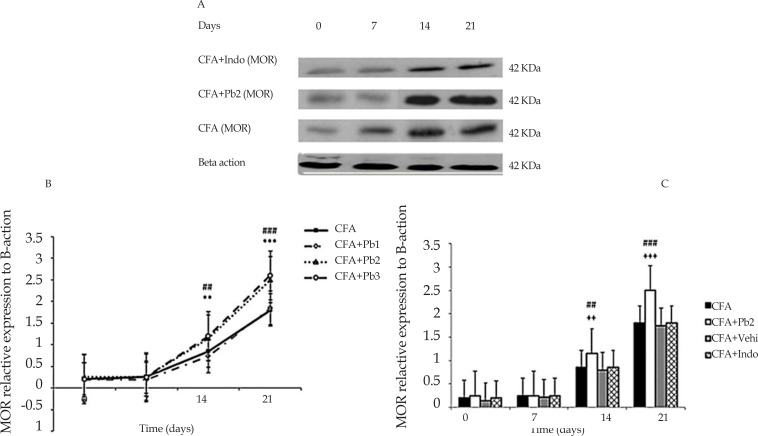
MOR expression A. Immunoblots of spinal MOR expression throughout different stages of arthritis. All data are represented as Mean±SEM (n=6/group). B. Administration of different doses of probiotics could affect MOR/β-actin ratio. Results were expressed as Mean±SEM and n=6/group. ^**^ P≤0.01 and ^***^ P≤0.001: Comparison of spinal MOR protein band intensity in different days of the study compared with baseline in the CFA group. ## P≤0.01 and ### P≤0.001: The differences in the MOR expression in the CFA+Pb2 (1/500) group on days 7, 14 and 21 compared with the same days in the CFA group. C. Administration of different doses of probiotics could increase MOR/β-actin ratio. Results were expressed as Mean±SEM and n=6/group. ^**^ P≤0.01 and ^***^ P≤0.001: Comparison of MOR/β-actin ratios between different days of the study in CFA+Indo group with CFA group. ++ P≤0.01; and +++ P≤0.001: Comparison of MOR/β-actin ratios between different days of the study in CFA+Indo group with CFA+Pb2 (1/500) group.

Injection of sterile mineral oil to the rat’s hind paw in the CFA control group did not cause noticeable variations in the spinal Pp38/p38MAPK activity compared to the baseline in different time points (graphing results not presented) (Figure 5A). Furthermore, oral administration of probiotics with a dose of 1/250 had no remarkable effect on the MOR/β-actin expression in the CFA+Pb1 group compared to the CFA group in different time points. With 1/500 and 1/1000 doses, it considerably elevated the MOR/β-actin expression in the CFA+Pb2 and CFA+Pb3 groups compared to the CFA group on days 14 and 21 (P≤0.01 for day 14, and P≤0.001 for day 21). There was no apparent discrepancy in expression of MOR/β-actin between two groups received probiotics with doses of 1/500 and 1/1000. Accordingly, the probiotic dose of 1/500 was selected as the effective dose in this phase (Figure 5B).

Comparison between CFA+Pb2 and CFA+Indo groups indicated that the effective dose of probiotics (1/500) remarkably increased the MOR/β-actin expression on days 14 and 21 compared to the same days in the CFA+Indo group (P≤0.01 for day 14, and P≤0.001 for day 21). Furthermore, administration of indomethacin had no noticeable effect on the MOR/β-actin expression in the CFA+Indo group compared to the CFA group. Changes in the MOR/β-actin expression in CFA+Vehi group showed no significant difference compared to those in the CFA group (Figure 5C).

## 4. Discussion

The results of this study revealed the important role of effective dose of probiotics in reducing edema, hyperalgesia, serum levels of IL-1β, spinal p38MAPK activity; and increasing levels of MOR expression during different phases of arthritis caused by CFA adjuvant. Inflammation and edema in the hind paw were induced by plantar injection of CFA which continued up to day 21 after injection. Hyperalgesia significantly increased on the seventh day after the CFA injection, but considerably decreased on days 14 and 21 compared to that on day 7.

Activation of afferent pain fibers during arthritis, decrease in pain threshold, and increase in the intensity of supra-threshold painful stimuli are important factors in the hyperalgesia development (Treede, Meyer, Raja, & Campbell, 1992). Scientists have reported that hyperalgesia can be induced by plantar injection of CFA, which continues up to 21 days (Nazemian et al., 2016). Our results indicated that the CFA-injected paw responded by a rapid onset of inflammation one day after injection and continued and increased up to day 21. Chronic phase (i.e. arthritic phase) of arthritic inflammation (next two weeks) was shown as a progressive increase in the injected paw volume; however, hyperalgesia decreased considerably compared to the acute inflammatory phase (first week).

It is evident that the first phase of arthritis is accompanied with the release of mediators such as histamine and proinflammatory cytokines, particularly TNF-α and IL-1β (Vaghef-Mehrabany et al., 2014; Zaringhalam et al., 2016). Studies also have indicated the important role of IL-1β in induction of hyperalgesia via direct action on neurons and stimulation of pain receptors as well as joint inflammation and destruction (Zaringhalam et al., 2016, Cantagrel et al., 1999). Accordingly, in the current study, the arthritis caused by CFA injection significantly increased the serum levels of IL-1β in the different days of study up to 21 days.

Studies have shown that inflammatory cytokine antagonists have a direct effect on cytokine levels in their intracellular signaling process which subsequently alleviate inflammatory symptoms (Vaghef-Mehrabany et al., 2014; Suzuki et al., 2000). The immune-boosting effects of probiotic bacteria can increase the levels of anti-inflammatory cytokines and immunoglobulins, reduce the levels of proinflammatory cytokines, actuate macrophages, increase the activity of natural killer cells, and modulate immune responses (Shadnoush et al., 2013). Not surprisingly, the results of our study revealed that the edema, hyperalgesia and serum levels of IL-1β reduced after administration of an effective probiotic dose compared to the CFA group. In this regard, our results not only reported anti-inflammatory and effective role of probiotics during acute phase of CFA-induced arthritis, but also showed that continuing administration of probiotics with 1/500 and 1/1000 doses during arthritis could reduce inflammatory symptoms until the 21st day of study.

Some studies have suggested the momentous role of proinflammatory cytokines in induction of anti-inflammatory cytokines in chronic phase of arthritis. Therefore, inhibition of IL-1β during CFA-induced arthritis can exacerbate inflammatory symptoms in the chronic phase by inhibiting the secretion of anti-inflammatory cytokines (Akhtari, Zaringhalam, Rohani, & Tekieh, 2013). In our study, despite the low amount of IL-1β, anti-inflammatory effects of probiotics during chronic phase of CFA-induced arthritis were occurred via a pathway different from the direct inhibition of serum IL-1β pathway. Accumulating evidence indicates that probiotic bacteria are capable to downregulate the inflammatory factors of the immune system and increase regulatory and anti-inflammatory cytokines (Shadnoush et al., 2013; Zamani et al., 2016).

It has been reported that p38MAPK activation during arthritis can increase the production of proinflammatory cytokines, including IL-1β which can, in turn, strengthen the hyperalgesia as one of the inflammatory symptoms (Tekieh, Zaringhalam, & Akhtari, 2014). The diminution of phosphorylated p38MAPK (Pp38MAPK) following administration of probiotics revealed that probiotics inhibits upstream of p38MAPK activation and subsequently can reduce inflammation and pain (Thomas & Versalovic, 2010).

Our results indicated that active p38MAPK resulted from long-term administration of probiotics with effective dosage during the chronic phase of CFA-induced arthritis reduced along with a decrease in edema and hyperalgesia. It seems that diminution of p38MAPK activity can be considered as an anti-inflammatory mechanism due to long-term administration of probiotics. On the other hand, Moller and Villiger stated that although spinal p38MAPK activation following the production of proinflammatory cytokines (TNF-α and IL-1β) increased and caused the induction of rheumatoid arthritis, hyperalgesia reduced due to the higher opioid receptors expression in chronic phase of arthritis (Möller & Villiger, 2006).

Opioid receptors are capable of reducing intracellular cAMP levels by inhibiting adenylate cyclase, diminishing neuronal excitability via hyperpolarization caused by increased permeability of membrane to potassium, and lessening neurotransmitter release via inhibition of voltage-gated calcium channels. MORs are present in the dorsal horn in the lumbar segment of spinal cord where they process and relay afferent nociceptive signals to the central nervous system (Shadnoush et al., 2013; De schepper, Cremonini, & Park, Camilleri, 2004).

Due to the impact of probiotics on the expression of MORs and the role of these receptors in the betterment of inflammatory pain, it seems that probiotics partly apply some of their analgesic effects in this way. In this respect, the results of this study suggest that increased expression of spinal MORs resulted from the long-term oral administration of probiotic with effective dosage during CFA-induced arthritic inflammation, may reduce hyperalgesia during the chronic phase of CFA-induced arthritis.

The present findings not only amplified the pivotal role of IL-1β and p38MAPK in CFA-induced arthritis modulated by long-term probiotics use with effective dosage, but also suggested that MOR may have another effectual target for arthritis management via probiotic treatment. Moreover, the potent anti-edematogenic and anti-hyperalgesic effects, and mainly the anti-inflammatory effects of probiotics, raise the possibility that it can be a good candidate for the control of inflammatory pain compared to synthetic drugs like indomethacin. It should be noted that the most mechanisms that allow CNS to modulate peripheral inflammatory responses are still unidentified; hence, the anti-inflammatory pathways involved during long-term probiotic treatment need more investigations.

## Ethical Considerations

### Compliance with ethical guidelines

All study procedures were approved by the Ethics Committee for the use of animals in research studies. They followed the ethical guidelines for investigations of experimental acute and chronic pain in animals, and the ethical principles of the Declaration of Helsinki and National Institute of Health (NIH).
